# Discovery of novel small molecule inhibitors of S100P with *in vitro* anti-metastatic effects on pancreatic cancer cells

**DOI:** 10.1016/j.ejmech.2020.112621

**Published:** 2020-10-01

**Authors:** Ramatoulie Camara, Deborah Ogbeni, Lisa Gerstmann, Mehrnoosh Ostovar, Ellie Hurer, Mark Scott, Nasir G. Mahmoud, Tomasz Radon, Tatjana Crnogorac-Jurcevic, Pryank Patel, Louise S. Mackenzie, David Y.S. Chau, Stewart B. Kirton, Sharon Rossiter

**Affiliations:** a School of Life and Medical Sciences, University of Hertfordshire, Hatfield, AL10 9AB, UK; b Barts Cancer Institute, Queen Mary University of London, Charterhouse Square, London, EC1M 6BQ, UK; c School of Pharmacy and Biomolecular Sciences, University of Brighton, Brighton, BN2 4GJ, UK; d Eastman Dental Institute, University College London, 256 Grays Inn Rd, London, WC1X 8LD, UK

**Keywords:** Pancreatic cancer, Virtual screen, Inhibitor, Metastasis, Calcium-binding protein, S100P

## Abstract

S100P, a calcium-binding protein, is known to advance tumor progression and metastasis in pancreatic and several other cancers. Herein is described the *in silico* identification of a putative binding pocket of S100P to identify, synthesize and evaluate novel small molecules with the potential to selectively bind S100P and inhibit its activation of cell survival and metastatic pathways. The virtual screening of a drug-like database against the S100P model led to the identification of over 100 clusters of diverse scaffolds. A representative test set identified a number of structurally unrelated hits that inhibit S100P-RAGE interaction, measured by ELISA, and reduce *in vitro* cell invasion selectively in S100P-expressing pancreatic cancer cells at 10 μM. This study establishes a proof of concept in the potential for rational design of small molecule S100P inhibitors for drug candidate development.

## Highlights

• Virtual screening identified hits against S100P, a protein target involved in tumor progression and metastasis.
• *In vitro* ELISA screening identified 13 hits that inhibit S100P interaction with its extracellular binding target RAGE.
• Synthesis of a short analog series of hit **4** demonstrated proof of concept for inhibitor design and optimization.
• Active hits reduce *in vitro* cell invasiveness selectively in S100P-expressing pancreatic cancer cells.

## Graphical abstract

**Figure:**
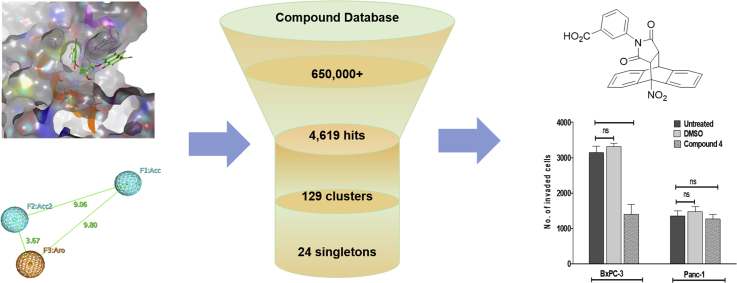

## 1 Introduction

Pancreatic cancer ranks as one of the most lethal cancers in the developed world. In the United States alone, it is estimated that during 2020 there will have been 57,600 diagnoses of pancreatic cancer and 47,050 deaths [1], and across Europe, it is estimated the 2018 disease incidence and mortality were 18.8 and 17.9 per 100,000 population respectively [2]. Despite the improvements in the survival rates of other cancers, there has been no major improvement in the survival rates for pancreatic cancer in the past 40 years, with still only around 5% of patients surviving the disease for ten years or more [3]. Pancreatic ductal adenocarcinoma (PDAC) represents the majority of pancreatic tumors and has the poorest prognosis.

Lack of early clinical markers and the largely asymptomatic nature of the disease contribute to late diagnosis, which is a major factor in the poor survival rate. Consequently, by the time of diagnosis, the vast majority of patients have advanced disease, for which chemotherapeutic interventions only offer a modest extension of lifespan at best, highlighting the urgent need for investigations into new therapies and new drug targets.

S100P is a calcium-binding protein that is highly expressed in pancreatic cancer in the early stages and in advanced, metastatic disease [4]. There has been much interest in its potential use as a prognostic or diagnostic marker [[5], [6], [7], [8]]. S100P has been shown to promote progression and metastasis in *in vivo* models of PDAC [9,10] and correlates with poor patient prognosis. S100P has also been reported in several other cancers, including colon, breast, lung, ovarian, nasopharyngeal and cervical cancers [11]. S100P binds the receptor for advanced glycation end-products (RAGE) extracellularly [12] and has a number of intracellular binding targets, including ezrin-palladin, integrin α7 and the not yet fully characterized S100P binding partner (S100PBP) [13], promoting a number of pathways for cell survival, proliferation, migration, and invasion [11].

The protein has shown some promise as a druggable target, with S100P’s cancer-promoting effects being suppressed through siRNA silencing [9] and small molecule downregulation of S100P expression [14]. Direct inhibition of S100P function has been achieved by use of a monoclonal antibody [15] and with the anti-allergy drug cromolyn **1** [16], indicating the possibility for the development of small molecules to directly target S100P. Cromolyn has been shown to bind to S100P and inhibit its binding to RAGE *in vitro*. It has also been shown to reduce metastatic load and to enhance sensitivity to the standard chemotherapeutic agent gemcitabine *in vivo* [16]. Cromolyn is, however, not likely to be a viable chemotherapeutic agent due to its low potency, lack of selectivity and low bioavailability. A series of cromolyn analogs have demonstrated some increased potency [17]. However, to date there has not been any reported progress in developing more potent and selective “drug-like” small molecule inhibitors of S100P, unrelated to cromolyn.

Virtual screening is an *in silico* methodology used in drug discovery and development projects to streamline and optimise candidate selection. It achieves this by exploiting computational models and algorithms which aim to accurately predict which molecules are likely to bind well to a biological target of therapeutic interest (and hence elicit an appropriate therapeutic response) [[18], [19], [20]].

Two experimental structures of S100P exist in the RCSB Protein Data Bank (PDB) as an X-ray crystal structure (PDB Accession Code 1J55) and an NMR ensemble (PDB Accession code 1OZO). The former is resolved as a 2 Å monomer with bound calcium ions but with residues 46–51 and 95 missing [21]. The NMR ensemble on the other hand contains 16 conformers that exist as dimers but with no bound calcium ions [22]. There are also three mutations in the ensemble compared to the native protein; T6→A, C85→S, and A92→T.

Using the available experimental information on cromolyn binding and the experimental S100P structures, this study employed *in silico* methods to identify potential binding pockets in the NMR ensemble of S100P, which could accommodate cromolyn, and to generate a pharmacophore model for S100P.

Subsequent virtual screening of lead-like databases identified hits – structurally distinct from cromolyn – that show promise as inhibitors of S100P’s tumor-promoting mechanisms and therefore potentially as chemotherapeutic agents for PDAC. Here, we report our generated pharmacophore, the results of the virtual screening of drug-like databases and the effects of selected hit compounds in protein and cell-based assays of S100P inhibition, and the associated functional effects.

## 2 Results and discussion

### 2.1 *In silico* modeling and virtual screening

**Fig. 1:**
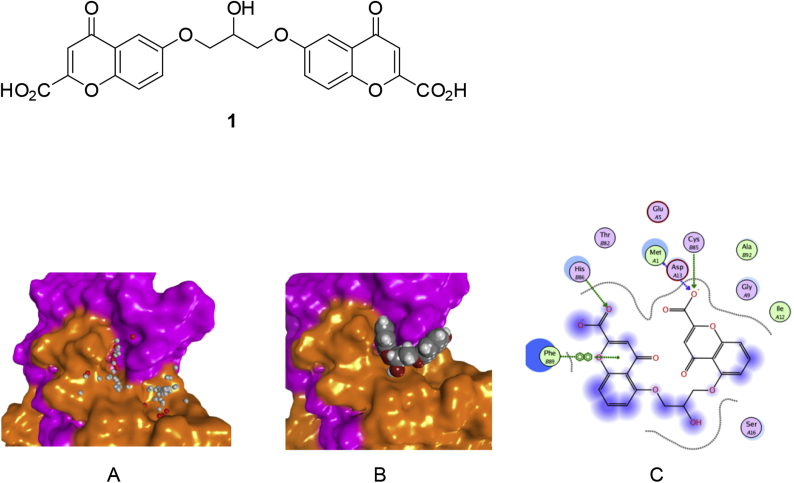
S100P interaction with cromolyn **1**. A: Two overlapping pockets identified at the dimeric interface of the protein (monomers in orange/pink) rendered with dummy atoms in MOE. B: Cromolyn (space-filling model) docked into pockets. C: Predicted binding interactions between cromolyn and S100P. (For interpretation of the references to colour in this figure legend, the reader is referred to the Web version of this article.)

Conformer number 15 in the NMR ensemble of S100P (1OZO) was identified as the most suitable structure for beginning drug discovery studies. Four different pocket detecting algorithms – Fpocket [23], Pocket-Finder [24], Q-SiteFinder [25] and MOE Site-Finder (Chemical Computing Group Inc.) [26] – independently identified a pocket at the S100P dimeric interface of this conformer that was large enough to bind cromolyn. This model coincidentally happens to be most representative model of 1OZO according to the authors who resolved the NMR ensemble [19]. The residues making up the pocket were located on both chains of the homodimer. Limitations of the pocket detection algorithms resulted in the pocket at the dimeric interface being resolved as two separate binding sites. The larger of the two pockets has a volume of 349 Å^3^ (Q-SiteFinder) with residues M1, T2, E5, T6, M8, G9, I12, F71, S72, and I75 from chain A, and F44, V78, A79, A80, I81, T82, S83, A84, C85, H86, K87, Y88, F89, K91, A92, G93, L94, and K95 from chain B contributing to the pocket surface. The smaller pocket is more buried than the first and has a volume of 198 Å^3^ with residues G9, I11, I12, D13, F15, S16, S19, S21, Q26, F71, S72, F74, I75, and V78 from chain A, and T82 and H86 from chain B contributing to the pocket surface (Fig. 1). F15, Y88 and F89 are involved in the hydrophobic core of S100P which, is exposed when the protein undergoes a conformational change upon calcium-ion binding [27]. The same pockets identified by the other programs were found to be sub-sets of those identified by Q-SiteFinder. In MOE Site-Finder, where identified cavities are ranked according to their propensity for ligand binding (PLB) score [28], the pockets identified at the dimeric interface were ranked in the top three with respect to propensity for ligand binding. In contrast to other published work on S100P-cromolyn interaction using the monomeric crystal structure, where it was reported that identified pockets on the protein were symmetrical [29], this was not observed in our study. Indeed, none of the pockets identified on the structures in the NMR ensemble show symmetricity. However, residues E5, D13, F44, Y88 and F89 of S100P that were previously identified to play a vital role in RAGE binding [30] were also identified in our pockets. Of note was the fact that the mutated residue A92 was present in the putative binding site identified by the independent pocket detection algorithms. To account for this, the structure was modified to return it to its native sequence. The effects of restoring the native amino acids on the structure was modeled by *in silico* site-directed mutagenesis and rotamer orientation optimization. There was no significant change to the binding pocket as a consequence of this.

When cromolyn **1** was docked into these pockets (Fig. 1B) using the Dock tool in MOE, the pose with the most interactions with the protein – three in total – had an estimated binding affinity energy score (S) of −10.54 kcal/mol. This pose had two hydrogen bond-interactions between the carboxylate oxygen at one end of the ligand and residue H86, and between the carboxylate oxygen at the other end with residue C85. The third interaction involved a pi-stacking hydrophobic interaction between residue F89 and one of the chromen-4-one moieties of the ligand (Fig. 1C).

These interactions served as the basis for the development of a final three-point pharmacophore model that was used in a virtual screen of lead-like databases in the MOE library. Combined, these databases had 653,214 compounds and the virtual screen returned 4619 hits – 0.71% of the databases. ChemAxon’s Library MCS clustering tool (JChem 5.9.0, 2013) available from ChemAxon (http://www.chemaxon.com) was used to cluster the hits based on their chemical similarity. There were 129 clusters with 24 singletons. The hit with the most negative binding affinity energy score (S) was selected from each cluster to give a diverse collection of hits with low energy scores. From this selection of 153 structures, a total of 68 representative database compounds were purchased, these being readily available from their commercial suppliers.

### 2.2 Initial biological screening of computational hits

The purchased computational screening hits were subjected to initial biological testing for inhibition of S100P-RAGE binding (ELISA), with cromolyn as positive control, using an adaptation of the reported ELISA for S100 family members [31].

**Fig. 2:**
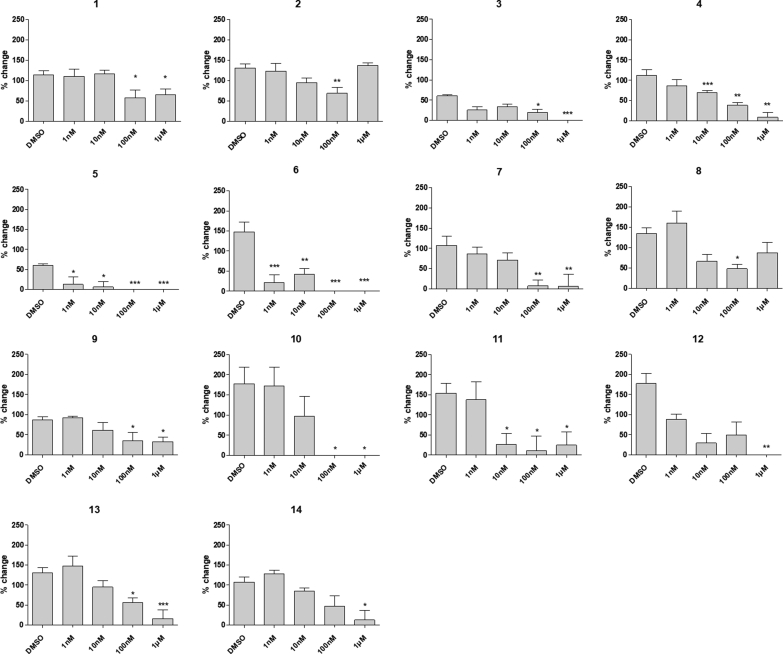
Effects of compounds **1**–**14** on S100P binding to RAGE. Human S100P (2 μM) coated wells were incubated with increasing concentrations of the compound tested, in triplicate, and exposed to human RAGE-Fc (100 nM) followed by goat anti-human secondary antibody to detect RAGE bound S100P. Data are compared to S100P/RAGE binding with no inhibitor (DMSO, 100%) and are presented as mean ± SEM; data is significant by one way ANOVA, where ∗ = p < 0.05, ∗∗ = p < 0.01 and ∗∗∗ = p < 0.001; n = 9 from 3 plates.

As expected, cromolyn inhibited S100P-RAGE binding at 100 nM and above in this assay, although it exhibited a maximum inhibition of only 56%, therefore a reliable estimate of IC_50_ could not be determined. Thirteen of the 68 diverse scaffolds tested (**2**–**14**, Table 1) exhibited significant activity in the ELISA at a concentration of 100 nM or lower (Fig. 2). Estimated IC_50_ values were calculated where sufficient % inhibition was observed in the assayed concentration range.

**Table 1:** Active hits, with estimated IC_50_ values, from virtual screening of the pharmacophore derived from the putative binding site of cromolyn, **1**, against MOE databases.

Compound	Vendor ID	Structure	Estimated IC_50_/nM
**2**	ChemBridge 7971920		ND
**3**	ChemBridge 7230553		0.514
**4**	ChemBridge 7356270		22.7
**5**	ChemBridge 7926943		0.600
**6**	Asinex ASN 06747799		0.555
**7**	Pharmex PHAR058776		19.0
**8**	Pharmex PHAR087402		ND
**9**	InterBioScreen STOCK2S-82643		7.70
**10**	InterBioScreen STOCK5S-26863		42.6
**11**	InterBioScreen STOCK5S-43994		6.89
**12**	InterBioScreen STOCK5S-58409		264
**13**	InterBioScreen STOCK5S-59895		126
**14**	InterBioScreen STOCK5S-33478		77.0

ND: not determined as maximum % inhibition insufficient to determine IC_50_.

**Fig. 3:**
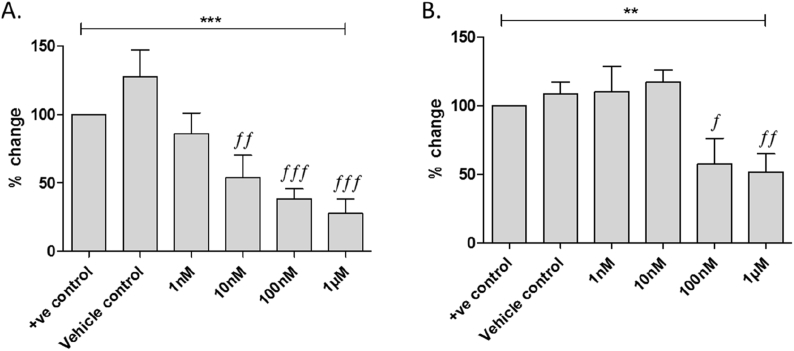
Compound **4** (A) and cromolyn **1** (B) block S100P binding to RAGE in a dose dependent manner. Human S100P (2 μM) coated wells were incubated with increasing concentrations of the compound tested, and exposed to human RAGE-Fc (100 nM) followed by goat anti-human secondary antibody to detect RAGE bound S100P. Data are compared to S100P/RAGE binding with no inhibitor (untreated, 100%) and 0.001% DMSO control (DMSO) and are presented as mean ± SEM; data is significant by one way ANOVA, where ∗∗ = p < 0.01 and ∗∗∗ = p < 0.001; significance by Dunnett’s Multiple comparison post-hoc test is expressed as *f* = p < 0.05, *ff* = p < 0.01 and *fff* = p < 0.001 compared to vehicle control (0.001% DMSO); n = 12 from 4 plates.

In the first screening round, compound **4** (ChemBridge Corp. San Diego, ID 7356270) exhibited an increased potency compared to cromolyn and significantly prevented S100P-RAGE interaction in a concentration-dependent manner at ≥10 nM (Fig. 3). Compound **4** has a molecular weight of 440 and cLogP of 2.80, which we considered a suitable starting point for analog synthesis, whilst retaining drug-like parameters (mol. wt. ≤500, cLogP ≤5.0); this structure was therefore selected as the first hit for further analog development.

### 2.3 Hit 4 analog synthesis

**Scheme 1:**
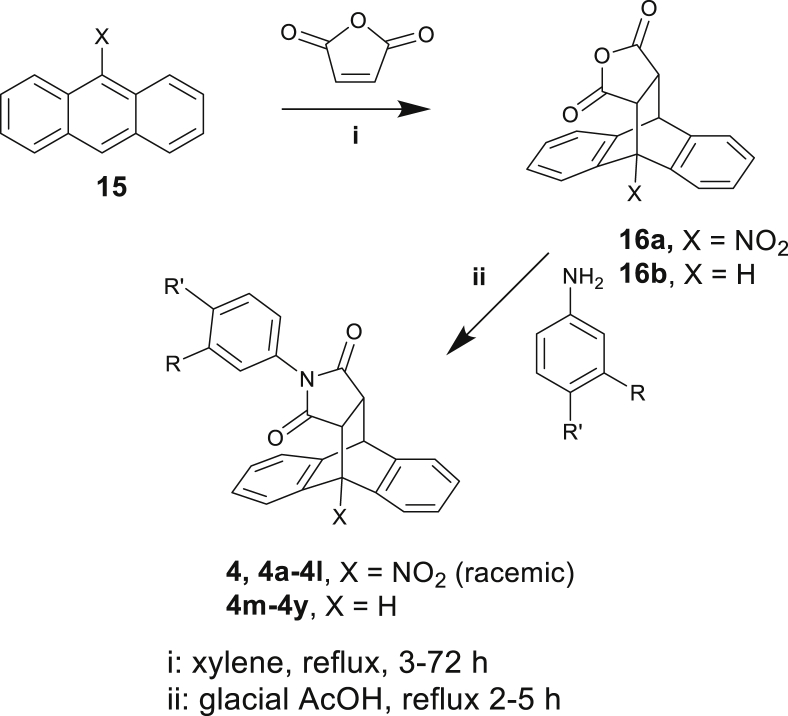
Synthesis of analogs of screening hit **4.**

Synthesis of compound **4** and a small series of analogs (**4a**-**4y**) was carried out using adaptations of published methods [32,33] (Scheme 1). From redocking of this hit it was decided to develop the library from solely 3- and 4- substituted anilines to explore the expected pharmacophore interactions, as *ortho*-substituents were a poorer fit for the pocket and the rotational restriction introduced could introduce a mixture of rotamers, as has been previously reported [34]. The series was designed to provide initial proof of concept that hits from the virtual screening could provide a valid scaffold for further hit-to-lead development [35].

A total of six analogs significantly inhibited S100P-RAGE interaction at or below 100 nM (**4a**, **4b**, **4f**, **4o**, **4r, 4u**), with others exhibiting weaker effects (Table 2).

**Table 2:** Initial analog series from hit compound **4**.

Compound	X	R	R′	Yield from 16a/b, %	ELISA inhibition	Estimated IC_50_/nM
**4**	NO_2_	CO_2_H	H	80	10 nM∗∗	22.7
**4a**	NO_2_	CO_2_Et	H	84	100 nM ∗∗∗	13.9
**4b**	NO_2_	F	H	81	100 nM∗	ND
**4c**	NO_2_	Cl	H	82	–	
**4d**	NO_2_	NO_2_	H	93	–	
**4e**	NO_2_	H	H	72	–	
**4f**	NO_2_	H	CO_2_H	80	<10 nM∗∗∗	0.482
**4g**	NO_2_	H	CO_2_Et	78	–	
**4h**	NO_2_	H	F	88	–	
**4i**	NO_2_	H	Cl	91	–	
**4j**	NO_2_	H	NO_2_	78	–	
**4k**	NO_2_	H	I	84	–	
**4l**	NO_2_	H	*t*-Bu	82	*-*	
**4m**	H	CO_2_H	H	95	–	
**4n**	H	CO_2_Et	H	96	–	
**4o**	H	F	H	87	10 nM∗	1.30
**4p**	H	Cl	H	92	–	
**4q**	H	NO_2_	H	95	–	
**4r**	H	H	H	83	10 nM∗	0.796
**4s**	H	H	CO_2_H	86	–	
**4t**	H	H	CO_2_Et	92	–	
**4u**	H	H	F	96	10 nM∗	4.15
**4v**	H	H	Cl	94	–	
**4w**	H	H	NO_2_	83	–	
**4x**	H	H	I	89	–	
**4y**	H	H	*t*-Bu	93	*-*	

Statistical significance by one-way ANOVA, ∗ = p < 0.05, ∗∗ = p < 0.01, ∗∗∗ = p < 0.001.

- = no significant inhibition below 1 μM, ND = not determined.

Selected positive hits from the assay (defined as those with significant reduction in S100P-RAGE binding at ≤1 μM) were then taken forward for cell functional assays. All compounds maintained solubility in 1% or 0.1% aqueous DMSO at the concentrations used in the assays.

### 2.4 Cell functional assays

#### 2.4.1 Effects of compounds on cell viability

To rule out general non-specific cytotoxicity or effects not specific to S100P-expressing cells, the effects on cell viability of five of the most active library compounds, **3**–**7**, plus the six analogs **4a**, **4b**, **4f**, **4o**, **4r** and **4u**, were assessed. Metabolic activity and cytotoxicity/cytolysis were measured with the MTS (CellTiter® 96 AQueous One Solution assay, Promega) and LDH release (CytoTox-ONE™ Homogeneous Membrane Integrity assay, Promega) assays respectively, in S100P-expressing (BxPC-3) and S100P-negative (Panc-1) cell lines, at a compound concentration of 10 μM. Cromolyn was used as a positive control.

**Fig. 4:**
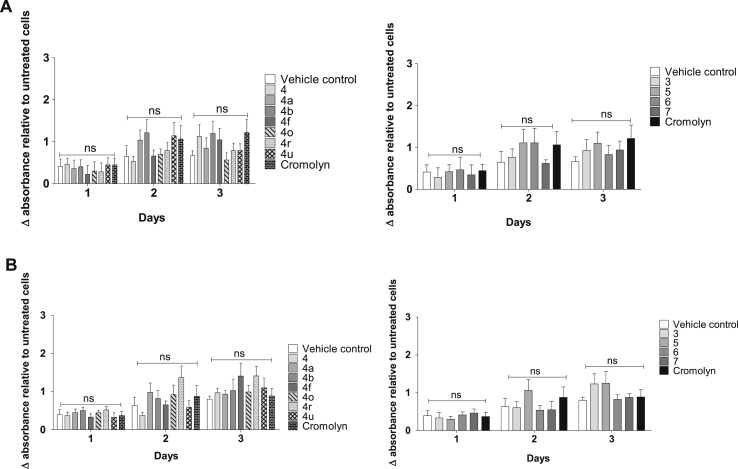
**A**. Effect of compounds on cell metabolic activity; 10^5^ BxPC-3 cells were exposed to 10 μM hit compounds for 24 h, and the effect on MTS staining for each time point measured by absorbance at 492 nm. Background media absorbance was subtracted from all values and expressed as a proportion of untreated cells. Data is expressed as mean ± SEM; data do not show any significant differences by two-way ANOVA; n = 3 separate experiments. **B:** Effect of compounds on cell metabolic activity of Panc-1 cells; 10^5^ Panc-1 cells were exposed to 10 μM hit compounds for 24 h, and the effect on MTS staining for each time point measured by absorbance at 492 nm. Background absorbance of media was subtracted from all values and expressed as a proportion of untreated cells. Data is expressed as mean ± SEM; data do not show any significant differences by two-way ANOVA; n = 3 separate experiments.

Samples were normalized to untreated cells. Viability and cytotoxicity were determined in comparison to the untreated control. Results indicate that the hit compounds have no significant effect on cell metabolic activity in either cell line, as measured by the MTS screen, compared to vehicle control (1% DMSO), over 3 days of incubation (Fig. 4A and B).

In a similar manner, data indicated that neither the hit compounds nor cromolyn exhibited cytotoxicity (measured *via* LDH release) over three days incubation in either cell-line, when compared to vehicle control (*Supplementary Data*).

#### 2.4.2 Effects of compounds on cell invasion

**Fig. 5:**
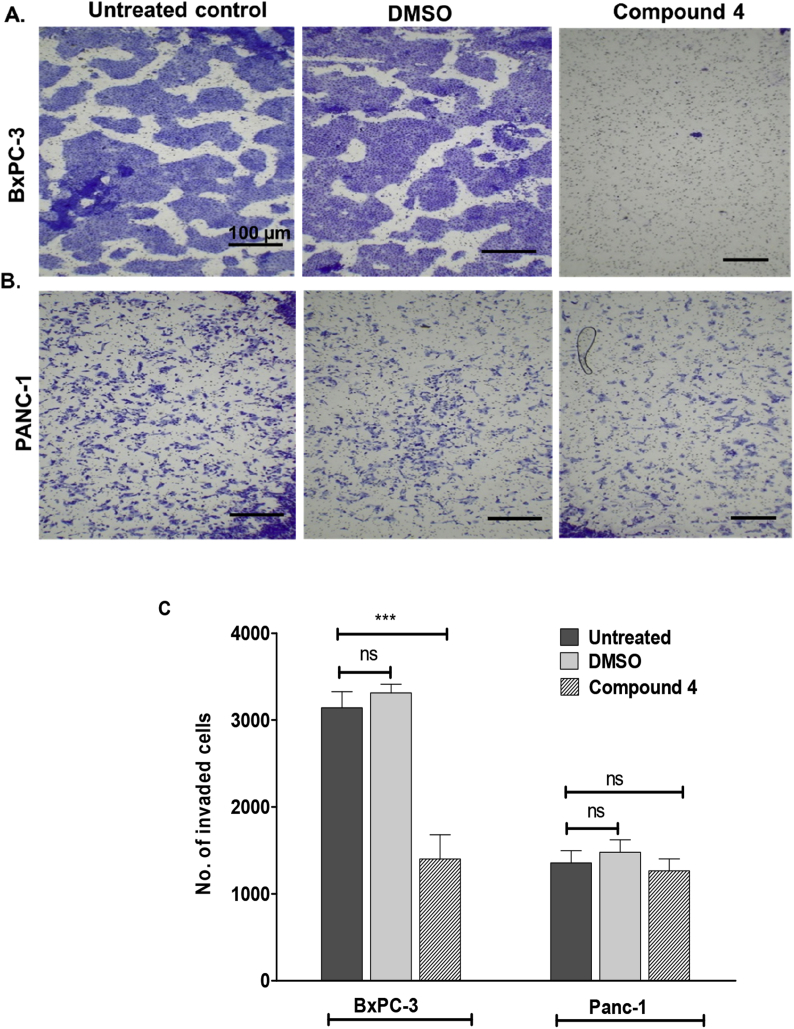
The invasive capacity of BxPC-3 and Panc-1 cells treated with 10 μM of compound **4** for 48 h. A) Invasion of BxPC-3 treated cells towards 10% FBS as a chemoattractant. B) Invasion of Panc-1 treated cells towards 10% FBS as a chemoattractant. C) Graphical representation of the data showing a comparison between the invasions of BxPC-3 and Panc-1 treated cells and controls. The total number of invading cells in the lower chamber was determined by counting the number of cells in 5 different fields, at 100x magnification. Error bars represent the mean ± standard error from three independent invasion experiments (n = 3; each containing average of 5 picture fields selected at x100 total magnification).

**Fig. 6:**
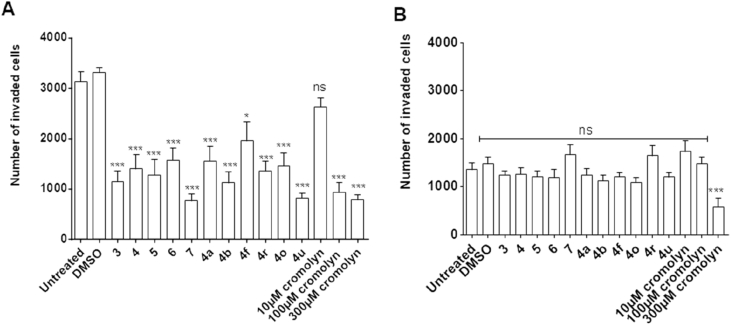
Transwell® invasion assay of A: BxPC-3 and B: Panc-1 cells treated with 10 μM hit compounds or cromolyn (10, 100, 200 μM); data are shown as the number of cells that have invaded through the matrix to the lower chamber. Data is shown as mean ± SEM number of invasive cells as counted under the microscope (x100). Significant difference between groups by one way ANOVA p < 0.001; Bonferroni’s post hoc test between cells exposed to chemoattractant (untreated) and DMSO (vehicle control, 0.001% DMSO) or cell exposed to hit compounds is indicated by ∗ = p < 0.05, ∗∗ = p < 0.01 and ∗∗∗ = p < 0.001; n = 10–15 observations from 3 separate experiments. N.B. Cromolyn exhibited significant inhibition of invasion at concentrations ≥100 μM.

The pro-metastatic effects promoted by S100P include an increase in cell migration and invasiveness. The effect of the S100P-binding hit compounds to inhibit cell invasion in S100P-expressing cells *in vitro* was measured *via* a Transwell invasion assay. In this assay, cells invade through the matrigel on upper Transwell chamber towards a lower chamber containing a chemoattractant. The results are shown in Fig. 5 and Fig. 6.

BxPC-3 cells (S100P-positive) exhibit a high invasiveness, as shown by the difference between negative control (no chemoattractant) and positive control (with chemoattractant). The vehicle control lacked any effect in blocking cell invasion, yet all of the selected ELISA-positive hit compounds were able to significantly (p < 0.001) inhibit invasion to the lower chamber at a concentration of 10 μM (Fig. 6). In this model, 10 μM cromolyn did not exhibit a statistically significant effect, although significant inhibition was observed at ≥100 μM. In comparison, Panc-1 cells, lacking S100P expression, exhibited a lower invasive trait, which was not significantly inhibited by any of the hit compounds.

Therefore, the observed reduction in cell invasiveness is consistent with an S100P-specific inhibitory effect.

Total protein content of invasive cells was measured by the BCA assay to confirm the reduced level of cells present. (*Supplementary Data*).

In order to exclude any possibility that reduced cell invasiveness was due to cell death during the time period of the assay, the viability of cells in the upper Transwell® chamber was assessed *via* measurement of LDH release. There was no significant increase in LDH release for either cell line after treatment with hit compounds or cromolyn. (*Supplementary Data*).

## 3 Conclusions

The model of the putative small molecule binding pocket of S100P, based upon the NMR structure of the apo-protein, has successfully led to identification of several new “drug-like” small molecule inhibitors of the S100P-RAGE interaction and S100P-mediated cell invasion *in vitro*. The compounds identified in this study represent a diverse set of scaffolds, providing proof of concept of the wider potential to design novel small molecule therapies that will have a functional and selective effect against S100P-expressing cancers. S100P inhibitors have potential to reduce metastatic spread and to be deployed in a combination therapy to improve the efficacy of standard chemotherapeutic treatments. There is an urgency in the search for therapeutic agents against pancreatic cancer, particularly in the management of advanced disease, which comprises the majority of new diagnoses. Therefore, this represents an exciting advance in the search for a targeted, novel therapy for PDAC and other S100P-expressing cancers.

## 4 Experimental

### 4.1 *In silico* modeling

#### 4.1.1 Preparation of experimental protein structures

The X-ray crystal structure of monomeric calcium bound S100P (1J55) was extracted from the PDB. The homodimer was generated from the deposited coordinates using the UCSF Chimera package [36][. Having generated the dimer all water molecules were removed from the structure. The NMR ensemble of S100P (1OZO) contains sixteen different conformations of homodimeric S100P. The ensemble was downloaded from the PDB website and the conformers were separated by copying their coordinates into Microsoft WordPad and saving as individual PDB files.

#### 4.1.2 Identification of potential binding pockets

Four complementary, yet independent, cavity detection algorithms were used to identify putative binding pockets at the dimeric interface for the experimental protein structures. A consensus approach was employed to minimise bias from a single pocket-detection algorithm, and hence identify the most credible cavities (i.e. those that spanned the dimeric interface of the protein and had sufficiently large volumes to accommodate cromolyn) for further study. The four algorithms used were: FPocket [23] (obtained from http://bioserv.rpbs.univ-paris-diderot.fr/cgi-bin/fpocket, currently available from https://github.com/Discngine/fpocket), Pocket-Finder [24] (obtained from http://www.modelling.leeds.ac.uk/pocketfinder/), Q-Site Finder [25] (obtained from http://www.modelling.leeds.ac.uk/qsitefinder/) and MOE Site Finder [26,37].

MOE SiteFinder algorithms used hypothetical hydrophilic probes of 1.4 Å radius, hypothetical hydrophobic probes of 1.8 Å and rejection criteria that excluded hydrophilic spheres with no hydrophobic alpha sphere within a radius of 3 Å.

*In silico* site-directed mutagenesis, and localised rotamer optimization, was carried out using the MOE software to return any mutated residues in the S100P NMR ensemble structures back to the native sequence.

#### 4.1.3 Docking studies – interaction of cromolyn at the S100P dimeric interface

The Dock algorithm in MOE was used to investigate the interactions between cromolyn and the putative binding pockets at the S100P dimeric interface. The cromolyn molecule was generated and minimised using the Molecule Builder application in MOE. Ionisation states at pH 7.4 were determined for cromolyn using the molecule washing function. The S100P protein file was protonated using Protonate 3D. The potential binding site identified at the dimeric interface using the pocket detection algorithms were recreated by selecting appropriate residues from the protein using the Sequence Editor Window and a surface map to be used in the docking was generated using a cut-off radius of 4.5 Å from the selected residues. The rotate bond option was switched on to allow different conformations of the cromolyn ligand to be examined during the docking run. The placement method used was triangle matcher, and the rescoring 1 function selected was London dG. Energy minimisation of the docked poses in the protein was carried out using the default Forcefield option in the Refinement dialogue box. The second refinement scoring function was set to “None”, which means that the final refined poses are ranked according to an MM/GBVI binding free energy estimate. Fifty poses were retained for each docking, and duplicate poses were removed from the analysis. The online server Ballaxy [38] was used to rescore high-ranked poses in an effort to eliminate scoring function bias. Any poses not re-scored highly were removed from further analysis.

### 4.2 Pharmacophore generation and validation

Pharmacophores were generated using the Pharmacophore Query Editor in MOE based on the results of the docking studies. Pharmacophore features highlighted by annotation points were selected in both the protein and the ligand. Default radii suggested by the Pharmacophore Query Editor were used. This gave rise to a four-point pharmacophore comprising an aromatic feature (Aro) and three anionic acceptor (Ani&Acc) interactions. This pharmacophore was employed in the virtual screening of the MOE lead-like database but matched only 52 of the 653,214 compounds (0.008% hit rate) in the database. This includes partial matches whereby three of the four pharmacophoric features were satisfied. It is possible for a single carboxylic acid moiety to satisfy two anionic acceptor features of the pharmacophore simultaneously (although in reality only one such H-bond interaction can occur at a time upon ligand-receptor binding), and hence the minimum number of an H-bond acceptors required for a hit to be considered a match from the virtual screening studies was one.

The four-point pharmacophore was very stringent. The distances between pharmacophoric features mean that any compounds returned by virtual screening would need to be relatively large. This coupled with the need for at least two acidic groups in each molecule to fulfil the anionic acceptor requirements explains the relatively low hit rate observed. In order to improve the hit-rate and increase the chemical diversity of the compounds identified by virtual screening, the pharmacophoric constraints were relaxed to reduce the number of constraints and modify the nature of the anionic acceptor requirement. By relaxing the constraints in terms of both the number of features that needed to be fulfilled and the type of acceptor functionality in the ligand, a three-point pharmacophore query was obtained that spanned the putative dimeric interface and consisted of two hydrogen bond acceptor features, and a hydrophobic feature.

The number of hits returned by the virtual screen with the relaxed pharmacophore was increased almost 100-fold (4619 hits, 0.71% hit rate). Cromolyn was seeded into the MOE-lead like database and was returned as a high-ranking hit using the refined pharmacophore. This gave some confidence that the tool was appropriately selective. It was not possible to validate the pharmacophore further, given that at the time of the study only cromolyn, and structurally similar derivatives, were shown to have activity against S100P. Hence, there was a paucity of molecules with known biological activities against S100P which could be used to further validate the pharmacophore.

#### 4.2.1 Screening database curation and filtering

The ‘lead-like’ compound from the ZINC database and the internal MOE screening database were used in the screen. Compounds from the ZINC database were imported into MOE as SMILES, converted into 3D molecules and “washed and filtered” prior to virtual screening taking place. This stage filtered out compounds with a molecular weight of >600, those compounds where the sum of hydrogen bond donors plus hydrogen bond acceptors was >12, those with more than seven rotatable bonds, those compounds with more than 4 chiral centres, compounds with more than 8 rings and any d-hybrid compounds.

Tanimoto coefficients were calculated for all remaining compounds in the database and one compound from those with pairwise values of 0.7 or greater was removed to ensure that a structurally diverse database, representative of lead-like chemical space but with the smallest number of compounds possible was created. These compounds were screened against the pharmacophores generated in the previous step.

#### 4.2.2 Hit clustering

Those compounds matching the pharmacophore were then clustered according to chemical structure using the ChemAxon software. The clustering employed was hierarchical, used a Maximum Common Substructure (MCS) value of 9 and used default matching parameters of all atom types, bond types, whole rings and charge. Medoids were identified from each cluster as potential compounds to take forward (subject to availability and resource) into the biological screening experiments.

### 4.3 General chemistry

Reagents for the chemical synthesis, unless specified otherwise, and anhydrous solvents were purchased from Sigma-Aldrich (Gillingham, Dorset, UK) and were used without further purification. All other solvents were purchased from Thermo Fisher Scientific (Loughborough, UK). Where necessary, solvents were dried using activated 3 Å (methanol, ethanol, dichloromethane) and 4 Å (diethyl ether) molecular sieves. ^1^H NMR and ^13^C NMR spectra were recorded on a JEOL ECA/54/SSS (400 or 600 MHz) spectrometer using TMS or residual solvent as internal standard. Deuterated solvents used for compound analysis are indicated with individual compound data. Raw NMR data files were processed with JEOL Delta 5.3.1. Chemical shifts are given in ppm relative to tetramethylsilane and *J* values (where given) are in Hz. Infrared spectra were recorded on a Scimitar 800 FT-IR spectrometer (Varian Inc.), a Nicolet 6700 FT-IR Smart iTR (Thermo Scientific) spectrometer using a Golden Gate™ Diamond ATR adapter, or a PerkinElmer FT-IR/FIR Spectrometer Frontier (Version 10.03.07), with samples prepared as thin films on the universal ATR sampling accessory. Liquid chromatography–mass spectrometry (LC-MS) was performed using a Varian 1200 L Quadrupole LC/MS/MS system equipped with Electrospray Ionisation (ESI) (Agilent Technologies, USA) in positive and negative ionisation modes and using a Varian Pursuit 50 mm × 4.6 mm 5 μm pore size C18 reverse phase column. Thin-layer chromatography (TLC) was carried out using Macherey-Nagel 60 Å (250 μm thick) flexible polyester sheet silica gel plates pre-coated with fluorescent indicator UV254. Dichloromethane was used as the mobile phase unless otherwise stated. Column chromatography was carried out using silica gel (high-purity grade, pore size 60 Å, 230–400 mesh particle size, 40–63 μm particle size, for flash chromatography, Sigma-Aldrich, Gillingham, Dorset, UK). Melting points were measured in open capillaries using a Griffin melting point apparatus and are uncorrected.

### 4.4 Synthesis of analogs of 4

#### 4.4.1 Dihydroanthracene Diels-Alder adducts **16a**, **16b**

Maleic anhydride (1.00 g, 10 mmol) was added to a stirred solution of anthracene (10 mmol mmol) in xylene (25 mL) and the mixture heated under reflux at 140–143 °C until all the diene disappeared, as indicated by TLC (3–72 h) [32]. The reaction mixture was cooled to room temperature before placing on ice with stirring. A precipitate separated out upon cooling, which was filtered, washed with ice-cold xylene and dried under suction to give the desired Diels-Alder cycloadduct as a pale-yellow powder which was used in the next step without further purification.

#### 4.4.2 *9-Nitro-9,10-dihydro-9,10-[3,4]furanoanthracene-12,14-dione (****16a****)*

Yield 65%; mp. 239–240 °C (lit. 244–245 °C [32]); ^1^H NMR (600 MHz, DMSO‑*d*_6_) *δ* (ppm) 7.67 (1H, m), 7.50–7.58 (2H, m), 7.44–7.37 (3H, m), 7.37–7.32 (1H, m), 7.05 (1H, dd, *J* = 7.5, 0.5 Hz), 5.11 (1H, d, *J* = 3.0 Hz), 4.66 (1H, d, *J* = 9.3 Hz), 3.94 (1H, dd, *J* = 9.3, 3.2 Hz); ^13^C NMR (150 MHz, DMSO‑*d*_6_) *δ* (ppm) 170.32, 168.99, 139.47, 137.17, 136.93, 134.15, 129.49, 129.44, 128.40, 128.17, 126.11, 125.60, 123.37, 120.81, 93.24, 51.14, 49.25, 44.79; IR *ν*_max_/cm^−1^: 2988, 2969, 1781, 1556, 1361; MS (ESI) found *m/z* 322.3 [M +H]^+^, C_18_H_11_NO_5_ calculated 321.1.

#### 4.4.3 *9,10-Dihydro-9,10-[3,4]furanoanthracene-12,14-dione (****16b****)*

Yield 92%, mp. 259–260 °C (lit. 261–263 °C [32]); ^1^H NMR (400 MHz, acetone-*d*_*6*_) *δ*.

(ppm) 7.51–7.47 (2H, m, Ar-CH), 7.38–7.33 (2H, m), 7.17–7.22 (4H, m), 4.91–4.89 (2H, m), 3.75–3.73 (2H, m); ^13^C NMR (100 MHz, acetone-*d*_*6*_) *δ* (ppm) 171.22, 141.51, 139.41, 127.23, 126.79, 125.09, 124.48, 48.24, 45.29; IR *ν*_max_/cm^−1^: 3077, 3026, 2970, 1782; MS (ESI) found *m/z* 277.1 [M +H]^+^, C_18_H_12_O_3_ requires 276.3.

#### 4.4.4 Synthesis of 13-(aryl)-9,10-dihydro-9,10-[3,4]epipyrroloanthracene-12,14-diones (**4**, **4a-4y**)

Maleic anhydride-anthracene cycloadduct **16a** or **16b** (0.3–0.5 mmol), and the appropriate 3- or 4-substituted aniline (1.2–1.5 eq) were stirred under reflux in glacial acetic acid (10 mL) between 1 and 5 h then cooled to room temperature. De-ionized water (25–40 mL) was added to the reaction mixture and the resulting precipitate was filtered, washed with ice-cold de-ionized water and dried. Recrystallization from glacial acetic acid furnished the products as white or cream crystalline solids.

#### 4.4.5 3-(9-Nitro-12,14-dioxo-9,10-dihydro-9,10-[3,4]epipyrroloanthracen-13-yl)benzoic acid (**4**)

Yield 80%; mp 271–272 °C; ^1^H NMR (600 MHz, acetone-*d*_6_) *δ* (ppm) 8.00–7.92 (1H, m), 7.77–7.69 (2H, m), 7.52–7.35 (7H, m), 7.27 (1H, dt, *J* = 5.2, 1.7 Hz), 7.13–7.06 (1H, m), 6.80–6.72 (1H, m), 5.03 (1H, d, *J* = 3.1 Hz), 4.58 (1H, d, *J* = 8.9 Hz), 3.72 (1H, dd, *J* = 8.9, 3.1 Hz). ^13^C NMR (150 MHz, DMSO‑*d*_6_) *δ* (ppm) 174.22, 173.02, 166.09, 139.36, 137.06, 136.90, 133.67, 131.79, 131.46, 130.58, 129.50, 129.39, 128.68, 128.38, 127.42, 127.35, 125.34, 124.84, 123.18, 120.15, 93.18, 49.08, 47.46, 44.72; IR *ν*_max_/cm^−1^: 3535 (br), 1705, 1551, 1366; MS (ESI, negative) found *m/z*: 439.2 [M − H]^-^, C_25_H_16_N_2_O_6_ calculated 440.1.

#### 4.4.6 *Ethyl 3-(9-nitro-12,14-dioxo-9,10-dihydro-9,10-[3,4]epipyrroloanthracen-*13-yl*)benzoate***(4a)**

Yield 84%; mp 202–203 °C; ^1^H NMR (600 MHz, DMSO‑*d*_6_) *δ* (ppm) 7.90 (1H, m), 7.70 (1H, d, *J* = 7.2 Hz), 7.65–7.63 (1H, m), 7.53–7.35 (6H, m), 7.05 (1H, d, *J* = 7.2 Hz), 6.99 (1H, t, *J* = 1.7 Hz), 6.77–6.75 (1H, m), 5.07 (1H, d, *J* = 3.1 Hz), 4.49 (1H, d, *J* = 8.6 Hz), 4.30 (2H, q, *J* = 7.2 Hz), 3.68 (1H, dd, *J* = 8.6, 3.1 Hz), 1.32 (3H, t, *J* = 7.2 Hz); ^13^C NMR (150 MHz, DMSO‑*d*_6_) *δ* (ppm) 174.22, 173.03, 164.49, 139.28, 137.08, 136.86, 133.68, 131.59, 131.18, 130.80, 129.67, 129.45, 128.68, 128.38, 127.42, 127.34, 127.21, 125.35, 124.85, 123.19, 120.15, 93.18, 61.11, 49.12, 47.50, 44.72, 14.07; IR *ν*_max_/cm^−1^: 2984, 2903 (CH stretch), 1710 (C=O stretch), 1551 (NO_2_ asymmetric), 1363 (NO_2_ symmetric); MS (ESI) found *m/z*: 469.3 [M + H]^+^. C_27_H_20_N_2_O_6_ calculated 468.1.

#### 4.4.7 *13-(3-fluorophenyl)-9-nitro-9,10-dihydro-9,10-[3,4]epipyrroloanthracene-12,14-dione (****4b****)*

Yield 81%; mp 241–243 °C; ^1^H NMR (600 MHz, DMSO‑*d*_6_) *δ* (ppm) 7.74 (1H, d, *J* = 7.6 Hz), 7.64–7.62 (1H, m), 7.49–7.47 (1H, m) 7.42–7.34 (5H, m), 7.24–7.20 (1H, m), 7.05 (1H, d, *J* = 7.6 Hz), 6.33–6.27 (2H, m), 5.06 (1H, d, *J* = 3.1 Hz), 4.49 (1H, d, *J* = 8.9 Hz), 3.56 (1H, dd, *J* = 8.9, 3.1 Hz); ^13^C NMR (150 MHz, DMSO‑*d*_6_) *δ* (ppm) 174.02, 172.82, 161.56 (d, ^*1*^*J*_C-F_ = 242 Hz), 139.28, 137.07, 136.85, 133.69, 132.56, 130.84, 128.69, 128.41, 127.43, 127.36, 125.36, 124.85, 123.20, 122.66, 120.16, 115.89 (d, ^*2*^*J*_C-F_ = 20 Hz), 113.69 (d, ^*2*^*J*_C-F_ = 24 Hz), 93.17, 49.00, 47.38, 44.72; IR *ν*_max_/cm^−1^: 3075, 3040, 3015, 2973, 2890, 1706, 1551, 1383; MS (ESI) found *m/z*: 415.0 [M + H]^+^, C_24_H_15_FN_2_O_4_ calculated 414.1.

#### 4.4.8 *13-(3-chlorophenyl)-9-nitro-9,10-dihydro-9,10-[3,4]epipyrroloanthracene-12,14-dione (****4c****)*

Yield 82%; mp 255-230 °C; ^1^H NMR (600 MHz, DMSO‑*d*_6_) *δ* (ppm) 7.71 (1H, d, *J* = 7.2 Hz), 7.66–7.63 (1H, m), 7.51–7.48 (1H, m), 7.46–7.40 (5H, m), 7.37 (1H, td, *J* = 7.2, 1.8 Hz), 7.06 (1H, d, *J* = 6.9 Hz), 6.48–6.46 (1H, m), 5.08 (1H, d, *J* = 3.3 Hz), 4.50 (1H, d, *J* = 8.9 Hz), 3.70 (1H, dd, *J* = 8.9, 3.2 Hz);^13^C NMR (150 MHz, DMSO‑*d*_6_) *δ* (ppm) 174.09, 172.91, 139.27, 137.11, 136.85, 133.71, 133.01, 132.48, 130.82, 128.94, 128.73, 128.42, 127.46, 127.38, 126.28, 125.41, 125.27, 124.90, 123.24, 120.20, 93.18, 49.07, 47.44, 44.72; IR *ν*_max_/cm^−1^: 3115, 2981, 2966, 1717, 1547, 1383. MS (ESI) found *m/z*: 431.4 [^35^Cl M + H]^+^, C_24_H_15_ClN_2_O_4_ calculated 430.1.

#### 4.4.9 *9-Nitro-13-(3-nitrophenyl)-9,10-dihydro-9,10-[3,4]epipyrroloanthracene-12,14-dione (****4d****)*

Yield 93%; mp 240–242 °C^; 1^H NMR (600 MHz, DMSO‑*d*_6_) *δ* (ppm) 8.23 (1H, m), 7.72 (1H, d, *J* = 7.2 Hz), 7.70 (1H, d, *J* = 7.8 Hz), 7.66–7.64 (1H, m), 7.53–7.50 (1H, m), 7.45–7.41 (3H, m), 7.39–7.36 (2H, m), 7.08 (1H, d, *J* = 7.2 Hz), 6.95 (1H, dd, *J* = 8.8, 2.1 Hz), 5.11 (1H, d, *J* = 3.0 Hz), 4.53 (1H, d, *J* = 8.9 Hz), 3.76 (1H, dd, *J* = 8.9, 3.1 Hz); ^13^C NMR (150 MHz, CD_2_Cl_2_) *δ* (ppm) 173.55, 172.23, 148.41, 138.77, 136.96, 136.39, 133.86, 132.37, 132.01, 130.10, 128.96, 128.80, 127.93, 127.76, 125.41, 124.40, 123.93, 123.72, 121.67, 120.95, 93.42, 49.13, 47.88, 46.04; IR *ν*_max_/cm^−1^: 3094, 3080, 3049, 2976, 2971, 2959, 2887, 1709, 1548, 1529, 1341; MS (ESI) found *m/z*: 442.4 [M + H]^+^, C_24_H_15_N_3_O_6_ calculated 441.1.

#### 4.4.10 *9-Nitro-13-phenyl-9,10-dihydro-9,10-[3,4]epipyrroloanthracene-12,14-dione (****4e****)*

Yield 72%; mp 110–112 °C^; 1^H NMR (600 MHz, DMSO‑*d*_6_) *δ* (ppm) 7.69 (1H, d, *J* = 7.2 Hz), 7.64–7.61 (1H, m), 7.48–7.45 (1H, m), 7.42–7.38 (3H, m), 7.36–7.32 (4H, m), 7.04 (1H, d, *J* = 7.2 Hz), 6.44–6.41 (2H, m), 5.05 (1H, d, *J* = 3.4 Hz), 4.48 (1H, d, *J* = 8.9 Hz), 3.65 (1H, dd, *J* = 8.9, 3.4 Hz); ^13^C NMR (150 MHz, DMSO‑*d*_6_) 174.39, 173.17, 139.42, 137.12, 136.99, 133.72, 131.28, 129.01, 128.81, 128.71, 128.41, 127.44, 127.36, 126.42, 125.39, 124.86, 123.23, 120.17, 93.21, 48.98, 47.39, 44.73; IR *ν*_max_/cm^−1^: 3070, 2972, 2903, 1713, 1550, 1387; MS (ESI) found *m/z*: 397.5 [M + H]^+^. C_24_H_16_N_2_O_4_ calculated 396.1.

#### 4.4.11 *4-(9-Nitro-12,14-dioxo-9,10-dihydro-9,10-[3,4]epipyrroloanthracen-*13-yl*)benzoic acid (****4f****)*

Yield 80%; mp 344–345 °C; ^1^H NMR (600 MHz, DMSO‑*d*_6_) *δ* (ppm) 7.90 (2H, d, *J* = 8.4 Hz), 7.70 (1H, d, *J* = 8.4 Hz), 7.64–7.62, (1H, m), 7.48–7.46 (1H, m), 7.41–7.35 (4H, m), 7.04 (1H, d, *J* = 7.2 Hz), 6.762 (2H, d, *J* = 8.4 Hz), 5.07 (1H, d, *J* = 3.0 Hz), 4.50 (1H, d, *J* = 9.0 Hz), 3.76 (1H, dd, *J* = 9.0, 3.0 Hz); ^13^C NMR (150 MHz, DMSO‑*d*_6_) *δ* (ppm) 174.04, 172.84, 166.35, 139.33, 137.00, 136.89, 134.87, 133.62, 131.01, 129.92, 128.69, 128.46, 127.41, 127.40, 126.35, 125.33, 124.84, 123.16, 120.15, 93.18, 49.06, 47.45, 44.72; IR *ν*_max_/cm^−1^: 3284 (br), 3076, 2970, 2903, 1702, 1550, 1389. MS (ESI, negative) found *m/z*: 439.4 [M − H]^-^, C_25_H_16_N_2_O_6_ calculated 440.1.

#### 4.4.12 *Ethyl 4-(9-nitro-12,14-dioxo-9,10-dihydro-9,10-[3,4]epipyrroloanthracen-*13-yl*)benzoate (****4g****)*

Yield 78%; mp 255–260 °C^; 1^H NMR (600 MHz, DMSO‑*d*_6_) *δ* (ppm) 7.94 (2H, d, *J* = 8.3 Hz), 7.71 (1H, d, *J* = 7.6 Hz), 7.65–7.63 (1H, m), 7.51–7.48 (1H, m), 7.43–7.35 (4H, m), 7.06 (1H, d, *J* = 6.9 Hz), 6.67 (2H, d, *J* = 6.9 Hz), 5.09 (1H, d, *J* = 2.8 Hz), 4.51 (1H, d, *J* = 8.9 Hz), 4.30 (2H, q, *J* = 6.9 Hz), 3.71 (1H, dd, *J* = 8.9, 2.8 Hz), 1.30 (3H, t, *J* = 6.9 Hz); ^13^C NMR (150 MHz, DMSO‑*d*_6_) *δ* (ppm) 174.05, 172.86, 164.81, 139.32, 137.01, 136.89, 135.20, 133.63, 130.02, 129.84, 128.73, 128.50, 127.44, 126.54, 125.36, 124.88, 123.18, 120.18, 93.19, 61.02, 49.09, 47.48, 44.74, 14.08; IR *ν*_max_/cm^−1^: 2989, 1710, 1550, 1394; MS (ESI) found *m/z*: 469.3 [M + H]^+^, C_27_H_20_N_2_O_6_ calculated 468.1.

#### 4.4.13 *13-(4-fluorophenyl)-9-nitro-9,10-dihydro-9,10-[3,4]epipyrroloanthracene-12,14-dione (****4h****)*

Yield 88%; mp 223–225 °C^; 1^H NMR (600 MHz, DMSO- *d*_6_) *δ* (ppm) 7.71 (1H, d, *J* = 7.0 Hz), 7.65–7.63 (1H, m), 7.49–7.47 (1H, m), 7.44–7.35 (4H, m), 7.23 (2H, t, *J* = 9.0 Hz), 7.06 (1H, d, *J* = 8.4 Hz), 6.50–6.47 (2H, m), 5.07 (1H, d, *J* = 3.0 Hz), 4.49 (1H, d, *J* = 8.4 Hz), 3.68 (1H, dd, *J* = 8.4, 3.0 Hz). ^13^C NMR (150 MHz, DMSO- *d*_6_) *δ* (ppm) 174.36, 173.15, 161.65 (^*1*^*J*_C-F_ = 242 Hz), 139.37, 137.12, 136.93, 133.72, 128.73, 128.60, 128.55, 128.44, 127.46, 127.38, 125.38, 124.89, 123.23, 120.19, 116.12 (d, ^*2*^*J*_C-F_ = 23 Hz), 93.20, 48.99, 47.38, 44.72. IR *ν*_max_/cm^−1^: 2981, 2970, 2893, 1710, 1551, 1395; MS (ESI) found *m/z*: 415.5 [M + H]^+^, C_24_H_15_FN_2_O_4_ calculated 414.1.

#### 4.4.14 *13-(4-chlorophenyl)-9-nitro-9,10-dihydro-9,10-[3,4]epipyrroloanthracene-12,14-dione (****4i****)*

Yield 91%; mp 270–273 °C; ^1^H NMR (600 MHz, DMSO-) *δ* (ppm) 7.66 (1H, d, *J* = 7.8 Hz), 7.60–7.58 (1H, m), 7.44–7.42 (61H, m), 7.39 (2H, d, *J* = 8.9 Hz). 7.38–7.35 (3H, m), 7.31 (1H, dt, *J* = 7.8, 1.4 Hz), 7.01 (1H, d, *J* = 7.2 Hz), 6.47–6.45 (2H, d, *J* = 8.9 Hz), 5.03 (1H, d, *J* = 3.0 Hz), 4.45 (1H, d, *J* = 8.9 Hz), 3.64 (1H, dd, *J* = 8.9, 3.0 Hz); ^13^C NMR (150 MHz, DMSO- *d*_6_) *δ* (ppm) 174.23, 173.03, 139.36, 137.10, 136.93, 133.70, 133.45, 130.05, 129.22, 128.77, 128.49, 128.13, 127.50, 127.43, 125.40, 124.91, 123.23, 120.23, 93.22, 49.07, 47.46, 44.76; IR *ν*_max_/cm^−1^: 2981, 2971, 2887, 1709, 1551, 1389; MS (ESI) found *m/z*: 431.4 [^35^Cl M + H]^+^, C_24_H_15_ClN_2_O_4_ calculated 430.1.

#### 4.4.15 *9-Nitro-13-(4-nitrophenyl)-9,10-dihydro-9,10-[3,4]epipyrroloanthracene-12,14-dione (****4j****)*

Yield 78%; mp 316–318 °C; ^1^H NMR (600 MHz, DMSO‑*d*_6_) *δ* (ppm) 8.24 (2H, d, *J* = 8.4 Hz), 7.71 (1H, d, *J* = 7.2 Hz), 7.63–7.61 (1H, m), 7.49–7.47 (1H, m), 7.42–7.34 (4H, m), 7.06 (1H, d, *J* = 7.6 Hz), 6.82 (2H, d, *J* = 8.4 Hz), 5.09 (1H, d, *J* = 3.0 Hz), 4.52 (1H, d, *J* = 8.8 Hz), 3.73 (1H, dd, *J* = 8.8, 3.0 Hz); ^13^C NMR (150 MHz, DMSO‑*d*_6_) *δ* (ppm) 173.87, 172.65, 147.01, 139.25, 136.99, 136.82, 136.55, 133.60, 128.77, 128.56, 127.50, 127.32, 125.36, 124.93, 124.46, 123.16, 120.22, 93.19, 49.16, 47.51, 44.74; IR *ν*_max_/cm^−1^: 3121, 3087, 2978, 2855, 1712, 1551, 1521, 1344; LC-MS (ESI) found *m/z*: 442.5 [M + H]^+^, C_24_H_15_N_3_O_6_ calculated 441.1.

#### 4.4.16 13-(4-iodophenyl)-9-nitro-9,10-dihydro-9,10-[3,4]epipyrroloanthracene-12,14-dione (**4k**)

Yield 84%; mp 305–308 °C; ^1^H NMR (400 MHz, DMSO‑*d*_6_) *δ* (ppm) 7.74–7.70 (3H, m), 7.63–7.61 (1H, m), 7.47–7.32 (5H, m), 7.05 (1H, d, *J* = 7.5 Hz), 6.28 (2H, d, *J* = 7.2 Hz), 5.06 (1H, d, *J* = 3.1 Hz), 4.48 (1H, d, *J* = 8.8 Hz), 3.66 (1H, dd, *J* = 8.8, 3.1 Hz); ^13^C NMR (100 MHz, DMSO‑*d*_6_) *δ* (ppm) 174.65, 173.45, 139.89, 138.50, 137.60, 137.47, 134.20, 131.46, 129.28, 128.91, 127.94, 125.90, 125.41, 123.73, 120.73, 95.62, 93.74, 49.59, 47.97, 45.28; IR *ν*_max_/cm^−1^: 2968, 2901, 1706, 1550, 1387; MS (ESI) found *m/z*: 523.3 [M + H]^+^, C_24_H_15_IN_2_O_4_ calculated 522.0.

#### 4.4.17 *13-(4-(tert-butyl)phenyl)-9-nitro-9,10-dihydro-9,10-[3,4]epipyrroloanthracene-12,14-dione (****4l****)*

Yield 82%; mp 238–240 °C; ^1^H NMR (400 MHz, CD_2_Cl_2_) *δ* (ppm) 7.75–7.72 (1H, m), 7.55–7.52 (1H, m), 7.43–7.28 (9H, m), 7.12–7.09 (1H, d, *J* = 7.6 Hz), 6.38 (2H, d, *J* = 7.9 Hz), 4.92 (1H, d, *J* = 3.1 Hz), 4.43 (1H, d, *J* = 9.0 Hz), 3.53 (1H, dd, *J* = 9.0, 3.1 Hz), 1.26 (9H, s). ^13^C NMR (150 MHz, CD_2_Cl_2_) *δ* (ppm) 174.34, 172.92, 152.38, 139.09, 137.23, 136.57, 133.95, 128.82, 128.58, 128.42, 127.72, 127.61, 126.18, 125.90, 125.37, 124.34, 123.89, 120.86, 93.47, 48.98, 47.74, 45.97, 34.65, 30.92; IR *ν*_max_/cm^−1^: 2981, 2902, 2875, 1714, 1547, 1392; MS (ESI) found *m/z*: 453.5 [M + H]^+^, C_28_H_24_N_2_O_4_ calculated 452.2.

#### 4.4.18 *3-(12,14-Dioxo-9,10-dihydro-9,10-[3,4]epipyrroloanthracen-*13-yl*)benzoic acid (****4 m****)*

Yield 95%; mp 285–288 °C (lit. 288–290 °C [39]); ^1^H NMR (600 MHz, DMSO‑*d*_6_) *δ* (ppm) 7.89–7.87 (1H, m), 7.53–7.51 (2H, m), 7.48 (1H, t, *J* = 7.8 Hz), 7.33–7.31 (2H, m), 7.22–7.20 (4H, m), 7.10 (1H, m) 6.68 (1H, d, *J* = 8.4 Hz), 4.88 (2H, s), 3.44 (2H, s).; ^13^C NMR (150 MHz, DMSO‑*d*_6_) *δ* (ppm) 175.86, 166.26, 141.59, 139.25, 132.01, 131.81, 130.71, 129.24, 127.50, 126.67, 126.39, 124.79, 124.40, 46.77, 44.85; IR *ν*_max_/cm^−1^: 3321, 1704, 1594, 1385; MS (ESI, negative) found *m/z*:394.2 [M − H]^-^, C_25_H_17_NO_4_ calculated 395.1.

#### 4.4.19 Ethyl 3-(12,14-dioxo-9,10-dihydro-9,10-[3,4]epipyrroloanthracen-13-yl)benzoate (**4n**)

Yield 96%; mp 205–207 °C; ^1^H NMR (400 MHz, acetone-*d*_6_) *δ* (ppm) 7.93 (1H, d, *J* = 7.8 Hz), 7.53–7.50 (2H, m), 7.45 (1H, t, *J* = 7.9 Hz), 7.37–7.30 (2H, m), 7.26–7.16 (5H, m), 6.80–6.77 (1H, m), 4.89 (2H, s), 4.33 (2H, q, *J* = 7.1 Hz), 3.47 (2H, s), 1.36 (3H, t, *J* = 7.1 Hz); ^13^C NMR (150 MHz, acetone-*d*_6_) *δ* (ppm) 175.63, 164.90, 141.95, 139.66, 132.81, 131.39, 131.29, 129.12, 129.02, 127.83, 126.84, 126.61, 125.01, 124.37, 60.94, 47.24, 45.81, 13.73; IR *ν*_max_/cm^−1^: 3070, 3042, 2992, 1718, 1703; MS (ESI) found *m/z*: 424.4 [M + H]^+^, C_27_H_21_NO_4_ calculated 423.2.

#### 4.4.20 13-(3-fluorophenyl)-9,10-dihydro-9,10-[3,4]epipyrroloanthracene-12,14-dione (**4o**)

Yield 87%; mp 228–230 °C; ^1^H NMR (400 MHz, CD_2_Cl_2_) *δ* (ppm) 7.46–7.41 (2H, m), 7.35–7.17 (7H, m), 7.05–6.97 (1H, m), 6.33 (1H, d, *J* = 7.6 Hz), 6.24 (1H, dt, *J* = 9.2, 2.2 Hz), 4.87–4.86 (2H, m), 3.39–3.38 (2H, m); ^13^C NMR (100 MHz, CD_2_Cl_2_) *δ* (ppm) 175.67, 162.44 (d, ^1^*J*_C-F_ = 245 Hz), 141.38, 139.00, 133.08, 133.00 (d, ^3^*J*_C-F_ = 10 Hz), 130.30 (d, ^3^*J*_C-F_ = 10 Hz), 127.20, 126.84, 125.08, 124.41, 122.44, 115.76 (d, ^2^*J*_C-F_ = 21 Hz), 114.05 (d, ^2^*J*_C-F_ = 24 Hz), 47.13, 45.89; IR *ν*_max_/cm^−1^: 3083, 3041, 3020, 2974, 1702; MS (ESI) found *m/z*: 370.4 [M + H]^+^, C_24_H_16_FNO_2_ calculated 369.1.

#### 4.4.21 13-(3-chlorophenyl)-9,10-dihydro-9,10-[3,4]epipyrroloanthracene-12,14-dione (**4p**)

Yield 92%; mp 238–240 °C; ^1^H NMR (600 MHz, DMSO‑*d*_6_) *δ* (ppm) 7.52–7.50 (2H, m), 7.43–7.37 (2H, m), 7.34–7.30 (2H, m), 7.24–7.19 (4H, m), 6.48–6.49 (2H, m), 4.88 (2H, s), 3.43 (2H, m); ^13^C NMR (150 MHz, DMSO‑*d*_6_) *δ* (ppm) 175.69, 141.48, 139.29, 133.07, 132.90, 130.65, 128.58, 126.66, 126.42, 125.40, 124.82, 124.42, 46.72, 44.86; IR *ν*_max_/cm^−1^: 3077, 2960, 1708; MS (ESI) found *m/z*: 386.3 [^35^Cl M + H]^+^, C_24_H_16_ClNO_2_ calculated 385.1.

#### 4.4.22 13-(3-nitrophenyl)-9,10-dihydro-9,10-[3,4]epipyrroloanthracene-12,14-dione (**4q**)

Yield 95%; mp 282–285 °C (lit. 248 °C [39]); ^1^H NMR (400 MHz, CD_2_Cl_2_) *δ* (ppm) 8.15–8.11 (1H, m), 7.52–7.42 (3H, m), 7.38–7.31 (3H, m), 7.27–7.19 (4H, m), 6.92 (1H, m), 4.88 (2H, s), 3.43 (2H, s); ^13^C NMR (150 MHz, CD_2_Cl_2_) *δ* (ppm) 175.41, 148.40, 141.19, 138.89, 132.65, 132.54, 129.91, 127.30, 126.90, 125.09, 124.41, 123.36, 121.77, 47.21, 45.93; IR *ν*_max_/cm^−1^: 3081, 3048, 3011, 2957, 1710, 1530, 1341; MS (ESI) found *m/z*: 397.4 [M + H]^+^, C_24_H_16_N_2_O_4_ calculated 396.1.

#### 4.4.23 13-Phenyl-9,10-dihydro-9,10-[3,4]epipyrroloanthracene-12,14-dione (**4r**)

Yield 83%; mp 211–215 °C; ^1^H NMR (400 MHz, DMSO‑*d*_6_) *δ* (ppm) 7.52–7.50 (2H, m), 7.36–7.28 (5H, m), 7.23–7.19 (4H, m), 6.44–6.42 (2H, m), 4.86 (2H, s), 3.41 (2H, s); ^13^C NMR (150 MHz, DMSO‑*d*_6_) *δ* (ppm) 176.02, 141.66, 139.34, 131.85, 128.89, 128.53, 126.68, 126.59, 126.41, 124.83, 124.41, 46.66, 44.89; IR *ν*_max_/cm^−1^: 3069, 3038, 2969, 1710; MS (ESI) found *m/z*: 352.4 [M + H]^+^, C_24_H_17_NO_2_ calculated 351.1.

#### 4.4.24 4-(12,14-Dioxo-9,10-dihydro-9,10-[3,4]epipyrroloanthracen-13-yl)benzoic acid (**4s**)

Yield 86%; mp 354–355 °C; ^1^H NMR (600 MHz, DMSO‑*d*_6_) *δ* (ppm) 7.90 (2H, d, *J* = 8.4 Hz), 7.53–7.50 (2H, m), 7.33–7.30 (2H, m), 7.22–7.19 (4H, m), 6.63 (2H, d, *J* = 8.4 Hz), 4.88 (2H, s), 3.44 (2H, m); ^13^C NMR (150 MHz, DMSO‑*d*_6_) *δ* (ppm) 175.69, 166.49, 141.57, 139.23, 130.71, 129.86, 126.73, 126.47, 126.41, 124.79, 124.40, 46.74, 44.87; IR *ν*_max_/cm^−1^: 3283 (OH stretch), 3020 (CH stretch), 2972 (CH stretch), 1698 (C=O stretch), 1105 (C–O stretch); MS (ESI, negative) found *m/z*: 394.2 [M − H]^-^, C_25_H_17_NO_4_ calculated 395.1.

#### 4.4.25 Ethyl 4-(12,14-dioxo-9,10-dihydro-9,10-[3,4]epipyrroloanthracen-13-yl)benzoate (**4t**)

Yield 92%; mp 220–222 °C; ^1^H NMR (600 MHz, acetone-*d*_6_) *δ* (ppm) 7.91 (2H, d, *J* = 9.0 Hz), 7.51–7.49 (2H, m), 7.32–7.30 (2H, m), 7.21–7.18 (4H, m), 67.0 (2H, d, *J* = 9.0 Hz), 4.87 (2H, s), 4.30 (2H, q, *J* = 7.2 Hz), 3.45 (2H, s), 1.32 (3H, t, *J* = 7.2 Hz); ^13^C NMR (150 MHz, acetone-*d*_6_) *δ* (ppm) 175.42, 165.09, 141.95, 139.62, 129.65, 126.85, 126.67, 126.61, 124.99, 124.37, 60.86, 47.20, 45.82, 13.67; IR *ν*_max_/cm^−1^: 2954 (CH stretch), 1707 (C=O stretch); MS (ESI) found *m/z*: 424.5 [M + H]^+^, C_27_H_21_NO_4_ calculated 423.2.

#### 4.4.26 13-(4-fluorophenyl)-9,10-dihydro-9,10-[3,4]epipyrroloanthracene-12,14-dione (**4u**)

Yield 96%; mp 250–253 °C; ^1^H NMR (600 MHz, DMSO‑*d*_6_) *δ* (ppm) 7.51–7.49 (2H, m), 7.30–7.29 (2H, m), 7.21–7.17 (6H, m), 6.47–6.45 (2H, m), 4.85 (2H, m), 3.40 (2H, m), ^13^C NMR (150 MHz, DMSO‑*d*_6_) *δ* 175.85, 161.43 (d, ^1^*J*_C-F_ = 244 Hz), 141.53, 139.26, 128.60, 127.96, 126.59, 126.33, 124.74, 124.33, 115.84 (d, ^2^*J*_C-F_ = 23 Hz), 46.59, 44.81; IR *ν*_max_/cm^−1^: 2975, 2891, 1708; MS (ESI) found *m/z*: 370.1 [M + H]^+^, C_24_H_16_FNO_2_ calculated 369.1.

#### 4.4.27 13-(4-chlorophenyl)-9,10-dihydro-9,10-[3,4]epipyrroloanthracene-12,14-dione (**4v**)

Yield 94%; mp 275–278 °C; ^1^H NMR (400 MHz, CD_2_Cl_2_) *δ* (ppm) 7.52–7.50 (2H, m), 7.43 (2H, d, *J* = 9.0 Hz) 7.32–7.30 (2H, m), 7.21–7.19, (4H, m) 6.48 (2H, d, *J* = 9.0 Hz), 4.87 (2H, s), 3.41 (2H, m); ^13^C NMR (150 MHz, CD_2_Cl_2_) *δ* (ppm) 175.72, 141.38, 139.01, 134.41, 130.25, 129.17, 127.86, 127.14, 126.81, 125.05, 124.37, 47.11, 45.88; IR *ν*_max_/cm^−1^: 2981, 2973, 2885, 1702; MS (ESI) found *m/z*: 386.4 [^35^Cl M + H]^+^, C_24_H_16_ClNO_2_ calculated 385.1.

#### 4.4.28 *13-(4-nitrophenyl)-9,10-dihydro-9,10-[3,4]epipyrroloanthracene-12,14-dione (****4w****)*

Yield 83%; mp 294–295 °C; ^1^H NMR (600 MHz, DMSO‑*d*_6_) *δ* (ppm) 8.22 (2H, d, *J* = 9.0 Hz), 7.51–7.50 (2H, m), 7.31–7.30 (2H, m), 7.20–7.18 (4H, m), 6.81 (2H, d, *J* = 9.0 Hz), 4.88 (1H, s), 3.45 (2H, s); ^13^C NMR (150 MHz, DMSO‑*d*_6_) *δ* (ppm) 175.50, 146.79, 141.49, 139.20, 137.21, 127.34, 126.81, 126.48, 124.80, 124.47, 124.34, 46.84, 44.89; IR *ν*_max_/cm^−1^: 3121, 3082, 3017, 2973, 2860, 1708, 1522, 1344; MS (ESI) found *m/z*: 397.2 [M + H]^+^, C_24_H_16_N_2_O_4_ calculated 396.1.

#### 4.4.29 13-(4-iodophenyl)-9,10-dihydro-9,10-[3,4]epipyrroloanthracene-12,14-dione (**4x**)

Yield 89%; mp 303–307 °C; ^1^H NMR (400 MHz, DMSO‑*d*_6_) *δ* (ppm) 7.72 (2H, d, *J* = 8.4 Hz), 7.52–7.50 (2H, m), 7.31–7.29 (2H, m), 7.20–7.18 (4H, m), 6.27 (2H, d, *J* = 8.4 Hz), 4.86 (2H, s), 3.40 (2H, s); ^13^C NMR (100 MHz, DMSO‑*d*_6_) *δ* (ppm) 176.23, 142.13, 139.81, 138.35, 132.02, 129.04, 127.22, 126.96, 125.32, 124.94, 95.17, 47.24, 45.41; IR *ν*_max_/cm^−1^: 2973 (CH stretch), 1700 (C=O stretch); MS (ESI) found *m/z*: 478.4 ([M + H]^+^), C_24_H_16_INO_2_ calculated 477.0.

#### 4.4.30 13-(4-(*tert*-butyl)phenyl)-9,10-dihydro-9,10-[3,4]epipyrroloanthracene-12,14-dione (**4y**)

Yield 93%; mp 268–271 °C; ^1^H NMR (400 MHz, CD_2_Cl_2_) *δ* (ppm) 7.44–7.42 (2H, m), 7.33–7.29 (4H, m), 7.23–7.19 (4H, m), 6.37 (2H, d, *J* = 8.8 Hz), 4.84–4.86 (2H, m), 3.36 (2H, m), 1.26 (9H, s); ^13^C NMR (150 MHz, CD_2_Cl_2_) *δ* (ppm) 176.15, 151.98, 141.55, 139.10, 129.07, 127.07, 126.74, 126.08, 126.03, 125.06, 124.33, 47.11, 45.89, 34.60, 30.95; IR *ν*_max_/cm^−1^: 2963, 2903, 2868, 1707; MS (ESI) found *m/z*: 408.5 [M + H]^+^, C_28_H_25_NO_2_ calculated 407.2.

### 4.5 Biological assays

#### 4.5.1 Recombinant proteins

To generate human S100P recombinant protein, a pQE-30 (27.1 μg/μl) plasmid encoding S100P with N-terminal histidine tags (gifted by Dr Igor Barsukov, Liverpool University, UK), was transformed into BL21∗ competent *Escherichia coli* cells. Recombinant protein was purified using a HisTrap Nickel-nitrilotriacetic acid (Ni-NTA) agarose column (GE Healthcare, UK).

#### 4.5.2 ELISA-based binding assay

Purified recombinant S100P (2μM/well) in phosphate buffer (PBS; 137 mM NaCl, 2.7 mM KCl, 10 mM Na_2_HPO_4_, 1.8 mM KH_2_PO_4_, pH 7.4; supplemented with 1 mM CaCl_2_, 10 μM ZnCl_2_, and 0.5 mM MgCl_2_) was used for coating a 96 well plate (NunclonDelta, Fisher), and incubated overnight at 37 °C.

Following washing, S100P non-specific binding sites were blocked with 1% bovine serum albumin (BSA; Fisher, UK) in PBS for 2 h at 37 °C. Following three washes, S100P was incubated with increasing concentrations of compounds, in parallel to cromolyn and DMSO treated wells, all in triplicate. Then 30 nM recombinant human receptor for advanced glycation end products (rh/RAGE-Fc; R&D Systems) was added and incubated for 1 h at 37 °C. Controls included a negative control (S100P protein, no RAGE-Fc), a positive control (S100P protein plus RAGE), and vehicle control (0.001% DMSO in PBS) were included in each plate. After three washes in PBS, all wells were exposed to goat anti-human HRP conjugate (1:1000; Millipore) for 1 h at 37 °C in order to detect bound receptors. Subsequent to washes, wells were incubated for 20 min in the dark with 3,3′,5,5′-tetramethylbenzidene (TMB) substrate (Sigma, UK). Plates were read at 450 nm using a Multiskan Ascent spectrophotometer (Fisher Scientific, UK). All sample readings were normalized to the positive control (S100P plus RAGE-Fc). Statistical analysis was performed and graphs plotted using the GraphPad Prism software in the following way: One Way ANOVA was used to compare the effects of concentration of each compound to the effects of vehicle control (DMSO), and p values of <0.05 were considered statistically significant. A post hoc analysis was conducted using Dunnetts multiple comparisons test; results are indicated by ∗ = p < 0.05; ∗∗ = p < 0.01 and ∗∗∗ = p < 0.001.

#### 4.5.3 Cell culture: MTS cell viability and LDH cytotoxicity assay

Human pancreatic cancer cell lines PanC-1 (ATCC- CRL-1469), and BxPC-3 (ATCC-CRL-1687) were cultured in a humidified atmosphere 5% CO_2_ (MCO-18AIC (uv) Japan) in Dulbecco’s modified eagle’s medium (DMEM)-high glucose (D6429, Sigma, USA) and Roswell park memorial institute (RPMI)-1640 medium, respectively, supplemented with 10% fetal bovine serum (FBS) (R8758, Sigma, USA), 1% penicillin and streptomycin (Sigma, Israel) and 2 mM l-Glutamine (L-glut) (Gibco, UK).

1 × 10^5^ Panc-1 cells and 1x10^5^ BxPC-3 cells were plated and allowed to adhere for 24 h; the cells were treated with cromolyn or the hit compounds for up to 3 days. MTS (CellTiter® 96 AQueous One Solution assay, Promega) and LDH release (CytoTox-ONE™ Homogeneous Membrane Integrity assay, Promega) assays were carried out every 24 h for a total of 3 days. MTS data was calculated in the following manner: OD for background absorbance was subtracted and all samples normalized to untreated cells in order to ascertain whether any compound-dependent effect occurred.

Since formazan formation is proportional to LDH release, data was calculated by subtracting the OD background and plotting the mean ± SEM.

#### 4.5.4 Matrigel invasion assay

The Transwell invasion assay (Fisher Scientific, Germany) was performed using chambers with polyethylene terephthalate (PET) membrane filters with 8 μm pore size coated on the upper side with Matrigel (a layer of extracellular matrix (ECM) to mimic the process of the ECM invasion.

BxPC-3 and Panc-1 cells were cultured as described above for 24 h, were re-suspended and diluted in the appropriate serum-free medium to a seeding density of 2 × 10^5^ cells/mL and added to the upper chambers of the Transwell invasion chambers. DMEM and RPMI-1640 media were supplemented with 10% fetal bovine serum (FBS; Sigma, USA) to act as a chemoattractant.

Controls included untreated cells and vehicle controls cells treated with a relevant concentration of DMSO that were both exposed to a chemoattractant in the bottom chamber. All other cells were exposed to 10 μM hit compounds with chemoattractant in the bottom chamber, and incubated for 48 h at 37 °C in a humidified atmosphere 5% CO_2_.

Cells that had passed through the matrigel coated membrane to the lower surface were fixed and stained with Diff-Quik stain kit (Fisher, USA) according to the manufacturer’s instructions. The stained membranes were viewed with an inverted microscope (CKX41 Olympus, Japan x100). The number of invaded cells were counted in five different fields of view using the ImageJ 1.50i software (windows version of NIH Image, http://rsb.info.nih.gov/nih-image/).

Compounds were added to cells in the above assays as follows: 0.5 μL of a 10 mM stock solution in DMSO was added to the cell medium at a final volume of 0.5 mL, final compound concentration 10 μM, final DMSO concentration 0.1%.

## Declaration of competing interest

The authors declare that they have no known competing financial interests or personal relationships that could have appeared to influence the work reported in this paper.

## Appendix A Supplementary data

The following is the Supplementary data to this article:Biological data: ELISA data for active analogs of hit **4,** LDH cytotoxicity assays, BCA protein assay. ^1^H NMR and ^13^C NMR spectra for all synthesized compounds.
