# Supplementary material for: Discovery of novel small molecule inhibitors of S100P with in vitro anti-metastatic effects on pancreatic cancer cells
Source: Eur J Med Chem. 2020 Oct 1;203:112621. doi: 10.1016/j.ejmech.2020.112621 (PMC7501730; doi:10.1016/j.ejmech.2020.112621)
Supplement: Multimedia component 1 [file mmc1.docx]

Supplementary data for:

**Discovery of novel small molecule inhibitors of S100P, with *in vitro* anti-metastatic effects on pancreatic cancer cells**

Ramatoulie Camara^a^, Deborah Ogbeni ^a^, Lisa Gerstmann ^a^, Mehrnoosh Ostovar^a^, Ellie Hurer^a^, Mark Scott^a^, Nasir G. Mahmoud^b^, Tomasz Radon^b^, Tatjana Crnogorac-Jurcevic^b^, Pryank Patel^a^, Louise S. Mackenzie ^a,c^, David Y. S. Chau^a,d^, Stewart B. Kirton^a^, and Sharon Rossiter^a^*.

1. Page 2: BCA protein assay for invasive cells in Transwell® invasion assay. Experimental details and Figure S1
2. Page 3: Cell toxicity assay in Transwell ® invasion assay. Figure S2.
3. Pages 4-5: Cell toxicity assay in BxPC-3 and Panc-1 cells incubated with/without hit compounds. Figure S3a, S3b.
4. Page 6: ELISA for active hit analogues **4a**, **4b**, **4f**, **4o**, **4r**, **4u.** Figure S4
5. Pages 7-34: ^1^H and ^13^C NMR spectra for synthesised compounds.

Cell invasion assay. Total protein content for invaded cells on lower membrane of Transwell® chambers.

Bicinchoninic acid (BCA) protein assay

The total protein concentration of invasive cells on the lower surface of the Transwell® membrane was determined using the Pierce™ BCA protein assay Kit (Thermo Fisher Scientific, USA). To generate a standard protein curve, bovine serum albumin (BSA) protein (Fisher Scientific, UK) standards ranging in concentration from 0.2 to 1.0 mg/mL, were prepared to a final volume of 100 μL using a 2 mg/mL BSA stock (Fisher, UK). Then, 5 µL of each standard, were added into the wells of a Nunclon Delta surface 96-well plate (Fisher Scientific, Denmark) in triplicates.

The total protein concentration of cell lysates was determined from a standard curve which was plotted using the absorbance of the BSA standards measured at 620 nM using a Multiskan Ascent plate reader (Thermo Labsystems, USA). The protein standard curve was plotted after correction for the average standard blank (containing 0 mg/ml BSA) absorbance. Finally, the concentration of protein in the lysates was determined using the equation derived from the standard curve (y=mx+b) where y is the absorbance, x the unknown protein concentration and the constants m and b were obtained from the standard curve.


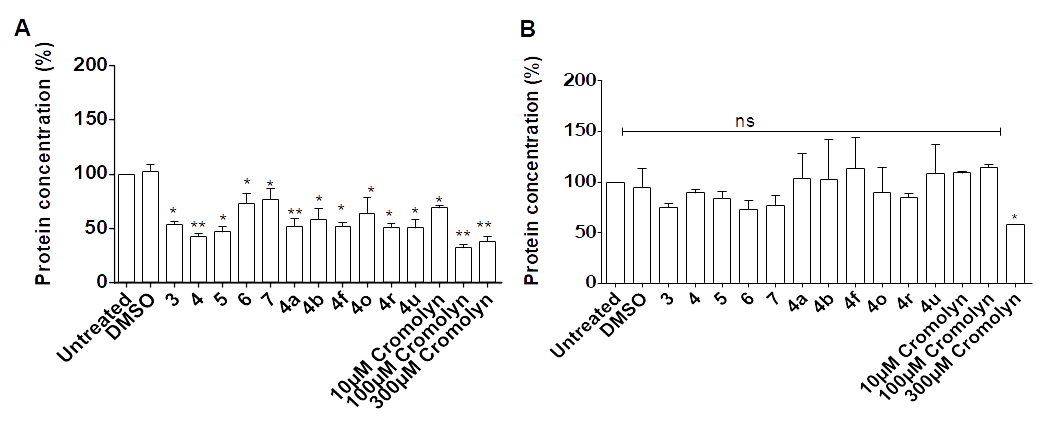


**Figure S1**. Protein concentration determined from BxPC-3 (**A**) and Panc-1 cells (**B**) on the Transwell® membrane after invasion assay. Stained invasive BxPC-3 and Panc-1 cells on Transwell® membrane were lysed, and the total protein concentration determined using BCA protein assay. Data represent the mean ± standard error of three independent experiments (n=3). The protein concentration was calculated as a percentage relative to the untreated control. Significance was assessed by One-way ANOVA, and Dunnett posthoc test **P<0.01, *P<0.05, (ns) P>0.05 in comparison to untreated control.

Cell toxicity assay: LDH release from cells remaining in upper Transwell® chamber.


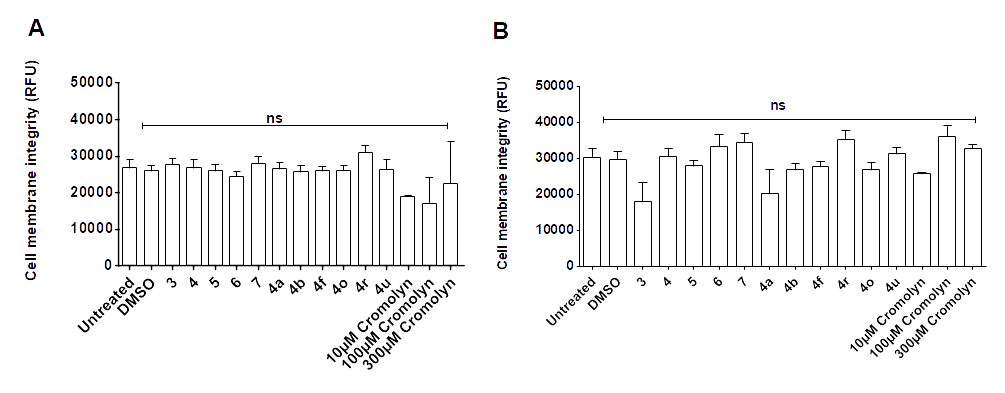


**Figure S2**: No significant difference in cell toxicity, measured by LDH release, is observed between treated and untreated cells in the Transwell® invasion assay. Cytotoxicity was measured using the medium recovered from the upper chamber of each Transwell ® chamber which contained BxPC-3 (**A**) and Panc-1 (**B**) cells treated with cromolyn (10 µM, 100 µM, and 300 µM) and hit compounds (10 µM) and incubated for 48 h. Data are the mean ± SEM of three independent experiments (n=3). One-way ANOVA assessed significance, and Dunnett post-hoc test is denoted by ns=P>0.05 in comparison to untreated control.

Cell toxicity on cells incubated for 3 days with hit compounds.


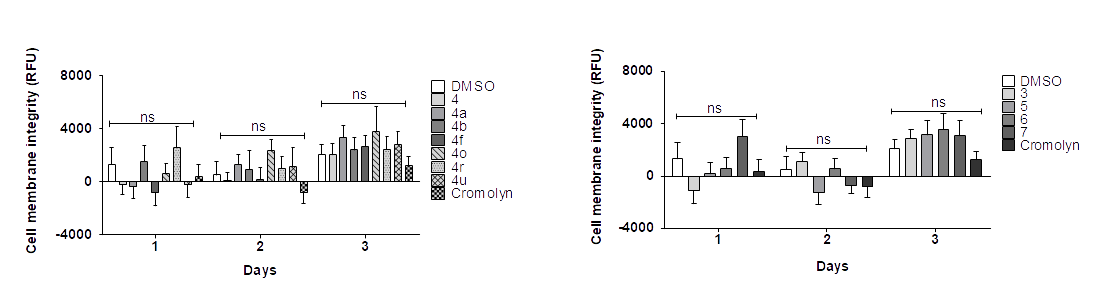


**Figure S3a**: Hit compounds show no significant cytotoxicity (determined by LDH release) in S100P-expressing BxPC-3 cells. 10^5^ BxPC3 cells were treated with 10 µM hit compounds for 24 hours, and the effect on LDH staining for each time point measured by fluorescence microplate reader. Background absorbance of media was subtracted from all values, and data is expressed as mean ± SEM; data do not show any significant differences by two way ANOVA; n=9 from 3 plates.


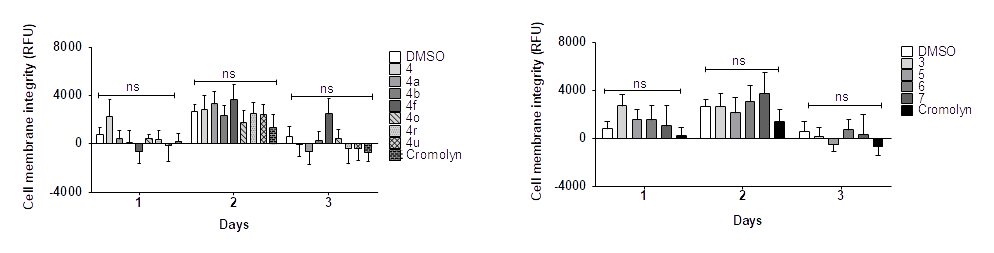


**Figure S3b**: Hit compounds show no significant cytotoxicity (determined by LDH release) in S100P-negative Panc-1 cells. as a measure of cytotoxicity. 10^5^ Panc- 1 cells were treated with 10 µM hit compounds for 24 hours, and the effect on LDH staining for each time point measured by fluorescence microplate reader. Background absorbance of media was subtracted from all values, and data is expressed as mean ± SEM; data do not show any significant differences by two way ANOVA; n=9 from 3 plates.

4. ELISA data for active hit analogues **4a**, **4b**, **4f**, **4o**, **4r**, **4u**

**Figure S4:** Effects of active hit analogues of compound **4** on S100P binding to RAGE. Human S100P (2 µM) coated wells were incubated with increasing concentrations of the compound tested, and exposed to human RAGE-Fc (100nM) followed by goat anti-human secondary antibody to detect RAGE bound S100P. Data are compared to S100P/RAGE binding with no inhibitor (DMSO, 100%) and are presented as mean ± SEM; data is significant by one way ANOVA, where * = p <0.05, **=p <0.01 and *** = p<0.001; n=12 from 4 plates.

5. NMR spectra for synthesised compounds.

Compound 16a

^1^H NMR spectrum

^13^C NMR spectrum

Compound 16b

^1^H NMR spectrum

^13^C NMR spectrum

Compound 4

^1^H NMR spectrum

^13^C NMR spectrum

Compound 4a

^1^H NMR spectrum

^13^C NMR spectrum

Compound 4b

^1^H NMR spectrum

^13^C NMR spectrum

Compound 4c

^1^H NMR spectrum

^13^C NMR spectrum

Compound 4d

^1^H NMR spectrum

^13^C NMR spectrum

Compound 4e

^1^H NMR spectrum

^13^C NMR spectrum

Compound 4f

^1^H NMR spectrum

^13^C NMR spectrum

Compound 4g

^1^H NMR spectrum

^13^C NMR spectrum

Compound 4h

^1^H NMR spectrum

^13^C NMR spectrum

Compound 4i

^1^H NMR spectrum

^13^C NMR spectrum

Compound 4j 1H NMR

^1^H NMR spectrum

^13^C NMR spectrum

Compound 4k

^1^H NMR spectrum

^13^C NMR spectrum

Compound 4l

^1^H NMR spectrum

^13^C NMR spectrum

Compound 4m

^1^H NMR spectrum

^13^C NMR spectrum

Compound 4n

^1^H NMR spectrum

^13^C NMR spectrum

Compound 4o

^1^H NMR spectrum

^13^C NMR spectrum

Compound 4p

^1^H NMR spectrum

^13^C NMR spectrum

Compound 4q

^1^H NMR spectrum

^13^C NMR spectrum

Compound 4r

^1^H NMR spectrum

^13^C NMR spectrum

Compound 4s

^1^H NMR spectrum

^13^C NMR spectrum

Compound 4t

^1^H NMR spectrum

^13^C NMR spectrum

Compound 4u

^1^H NMR spectrum

^13^C NMR spectrum

Compound 4v

^1^H NMR spectrum

^13^C NMR spectrum

Compound 4w

^1^H NMR spectrum

^13^C NMR spectrum

Compound 4x

^1^H NMR spectrum

^13^C NMR spectrum

Compound 4y

^1^H NMR spectrum

^13^C NMR spectrum
